# DDX3X coordinates host defense against influenza virus by activating the NLRP3 inflammasome and type I interferon response

**DOI:** 10.1016/j.jbc.2021.100579

**Published:** 2021-03-23

**Authors:** Sannula Kesavardhana, Parimal Samir, Min Zheng, R.K. Subbarao Malireddi, Rajendra Karki, Bhesh Raj Sharma, David E. Place, Benoit Briard, Peter Vogel, Thirumala-Devi Kanneganti

**Affiliations:** 1 Department of Immunology, St Jude Children’s Research Hospital, Memphis, Tennessee, USA; 2 Animal Resources Center and the Veterinary Pathology Core, St Jude Children’s Research Hospital, Memphis, Tennessee, USA

**Keywords:** DDX3X, influenza A virus, type I IFN, NLRP3, inflammasome, stress granule, NS1, immune evasion, innate immunity, host defense, ΔNS1, NS1 deletion mutant, ASC, apoptosis-associated speck-like protein containing a caspase recruitment domain, Ars, arsenite, BMDM, bone marrow–derived macrophage, CASP1, caspase-1, CASP3, caspase-3, CASP8, caspase-8, DDX3X, DEAD-box helicase 3 X-linked, G3BP1, GTPase-activating protein-binding protein 1, HRP, horseradish peroxidase, IAV, influenza A virus, iBMDMs, immortalized BMDMs, IFN, interferon, IL, interleukin, IRF1, interferon regulatory factor 1, MCMV, murine cytomegalovirus, MOI, multiplicity of infection, NLRP3, nucleotide-binding oligomerization domain-like receptor with a pyrin domain 3, NP, nucleoprotein, NS1, nonstructural protein 1, P-eIF2α, phosphorylation of eIF2α, P-IRF3, phosphorylation of IRF3, PR8, Puerto Rico/8/34, RIG-I, retinoic acid–inducible gene I, SG, stress granule, STAT1, signal transducer and activator of transcription 1, TLR, Toll-like receptor, VSV, vesicular stomatitis virus

## Abstract

Viruses and hosts have coevolved for millions of years, leading to the development of complex host–pathogen interactions. Influenza A virus (IAV) causes severe pulmonary pathology and is a recurrent threat to human health. Innate immune sensing of IAV triggers a complex chain of host responses. IAV has adapted to evade host defense mechanisms, and the host has coevolved to counteract these evasion strategies. However, the molecular mechanisms governing the balance between host defense and viral immune evasion is poorly understood. Here, we show that the host protein DEAD-box helicase 3 X-linked (DDX3X) is critical to orchestrate a multifaceted antiviral innate response during IAV infection, coordinating the activation of the nucleotide-binding oligomerization domain-like receptor with a pyrin domain 3 (NLRP3) inflammasome, assembly of stress granules, and type I interferon (IFN) responses. DDX3X activated the NLRP3 inflammasome in response to WT IAV, which carries the immune evasive nonstructural protein 1 (NS1). However, in the absence of NS1, DDX3X promoted the formation of stress granules that facilitated efficient activation of type I IFN signaling. Moreover, induction of DDX3X-containing stress granules by external stimuli after IAV infection led to increased type I IFN signaling, suggesting that NS1 actively inhibits stress granule–mediated host responses and DDX3X-mediated NLRP3 activation counteracts this action. Furthermore, the loss of DDX3X expression in myeloid cells caused severe pulmonary pathogenesis and morbidity in IAV-infected mice. Together, our findings show that DDX3X orchestrates alternate modes of innate host defense which are critical to fight against NS1-mediated immune evasion strategies during IAV infection.

Influenza virus infections have caused several pandemics and pose a constant threat to humans (1, 2, 3). Influenza A virus (IAV) infects primarily respiratory epithelial cells and causes pulmonary pathology; if uncontrolled, this infection leads to the loss of lung function and mortality (1, 2, 4). IAV infection is sensed by innate immune receptors that activate type I interferons (IFNs) and proinflammatory responses (5). These responses are critical for controlling viral titers and spread in the lung and also to induce long-lasting adaptive immune responses (5, 6). Acute activation of innate immune responses against IAV facilitates the recruitment of inflammatory immune cells including neutrophils and monocytes, which further establish the inflammatory milieu to clear virus-infected and dead cells to subsequently initiate lung tissue repair responses (5, 6). However, excessive innate immune activation, inflammatory cytokine secretion, and immune cell recruitment have been implicated in the morbidity and mortality of the 1918 influenza pandemic and also in recent highly pathogenic avian influenza (H5N1) infections (2, 7, 8, 9, 10). These observations indicate that robust but balanced activation of the innate immune response against IAV is crucial for host protective defense, whereas aggressive induction of these responses may compromise host lung function and ultimately lead to mortality. Thus, understanding the fundamental mechanisms orchestrating innate immune responses during IAV infection is critical to target IAV-induced pathogenesis and develop effective antiviral approaches.

Type I IFNs are primarily induced by sensing of viral RNAs by innate immune receptors, including retinoic acid–inducible gene I (RIG-I) and Toll-like receptors (TLRs), to exert host-induced antiviral activities (5, 11). In addition, activation of the nucleotide-binding oligomerization domain-like receptor with a pyrin domain 3 (NLRP3) inflammasome in IAV-infected host cells promotes recruitment of monocytes and neutrophils to the site of infection, facilitates clearance of virus-infected cells, and drives lung tissue repair responses (6, 12, 13, 14, 15, 16). Type I IFNs and inflammasome-dependent leaderless proinflammatory cytokines are the primary innate immune mediators which dominate acute host responses against IAV infection (5, 6, 8, 10, 11). Type I IFNs and the NLRP3 inflammasome responses are also key for activating adaptive immune responses, which play an essential role in controlling viral titers in later stages of infection and also subsequent IAV infections (6, 12, 13, 14, 17). Host defense is also mediated by ribonucleoprotein aggregates called stress granules (SGs) during IAV infection, which are formed in the absence of the IAV nonstructural protein 1 (NS1) (18). The antiviral activity of SGs is predominantly achieved through the restriction of translation or viral protein synthesis (19, 20, 21). Although type I IFNs, SGs, and the NLRP3 inflammasome are required for host defense responses during viral infections, the mechanisms regulating coordination of these responses to ultimately exert protection during IAV infection are not clear.

IAV has also evolved to use immune evasion strategies to neutralize protective host defense mechanisms (11, 22). NS1 of IAV antagonizes host defense mechanisms by interfering with type I IFN signaling and also disrupting formation of antiviral SGs (11, 22, 23, 24). The NS1-mediated host immune evasion suppresses antiviral defense mechanisms, which enables efficient replication of IAV (11, 22). NS1 is also implicated in regulating NLRP3 inflammasome activation, suggesting this protein has adapted to control a variety of innate immune activation mechanisms (25). It is possible that the IAV-mediated immune evasion strategies might have exerted selective pressure on the host immune system during virus–host interactions. This counter adaptation of host defense mechanisms may help fight viral immune evasion. However, the complex relationship between antiviral activities and their functional regulation in the presence of IAV-mediated immune evasion are not clear.

Here, we found a critical role for the host protein DEAD-box helicase 3 X-linked (DDX3X) in regulating a complex network of host defense responses by activating the NLRP3 inflammasome, formation of SGs, and type I IFNs during IAV infection. The IAV-induced SGs inhibited activation and assembly of the NLRP3 inflammasome. DDX3X performed two mutually exclusive functions, driving IAV-induced activation of the NLRP3 inflammasome or inducing the formation of SGs, and this function was dependent on the presence of the immune-evasive NS1 protein. Lack of DDX3X expression led to severe pathology and IAV spread in the lung, compromising survival in infected mice. Thus, DDX3X is a central regulator of host defense responses against IAV infection and perhaps evolved to counteract IAV immune evasions strategies.

## Results

### Inhibition of SGs and type I IFN response by NS1 promotes activation of the NLRP3 inflammasome

**Figure 1:**
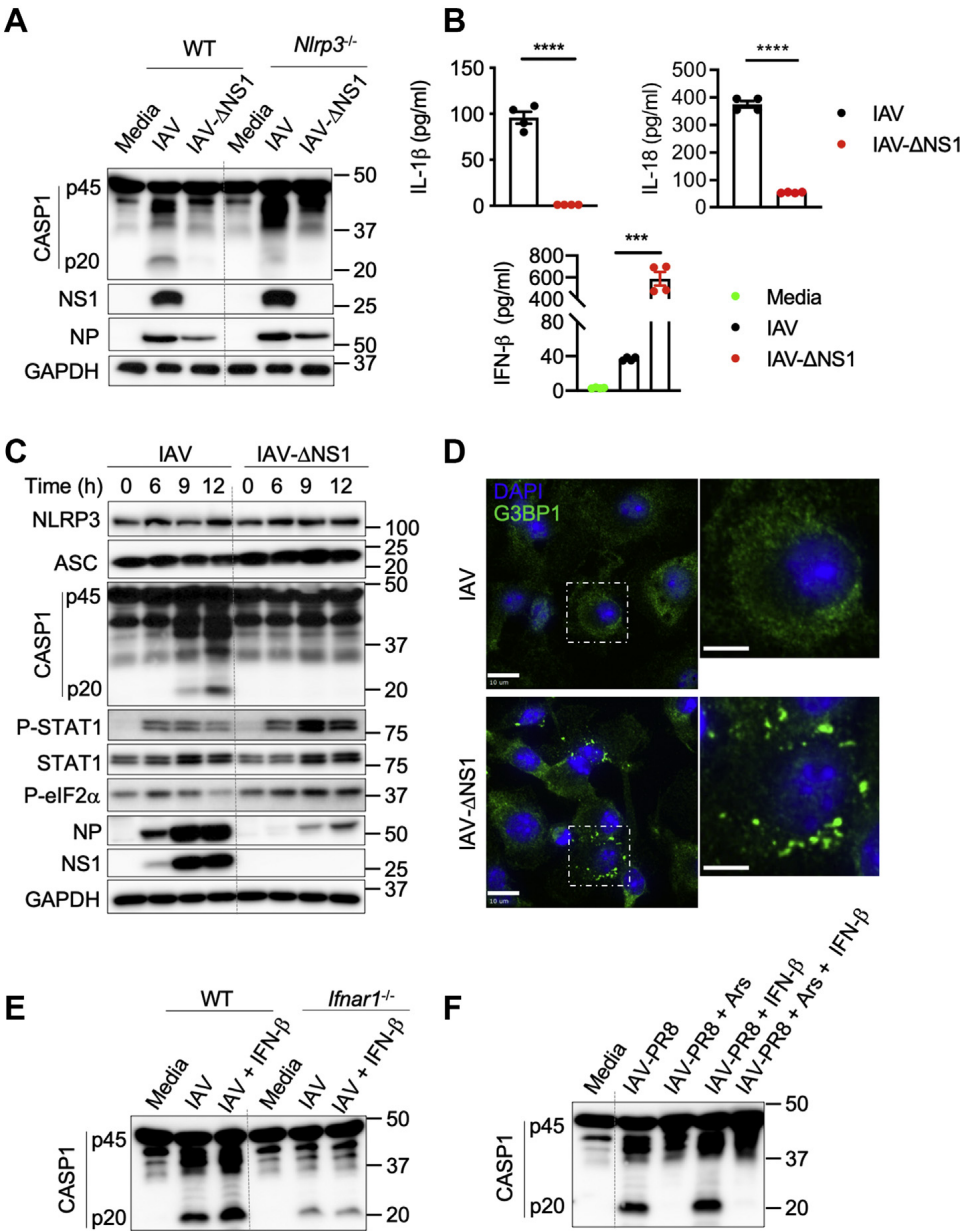
**Host antiviral immune responses in BMDMs are modulated based on the immune evasion potential of IAV.***A*, immunoblot analysis of caspase-1 (CASP1) cleavage (pro-CASP1 (p45) and cleaved CASP1 (p20)), levels of NS1, NP, and GAPDH in WT and *Nlrp3*^−/−^ bone marrow–derived macrophages (BMDMs) infected with influenza A virus (IAV) or IAV–ΔNS1 at MOI of 5. Representative blots (*n* = 4). *B*, ELISA measurement of IL-1β, IL-18, and IFN-β. ∗∗∗*p* = 0.001, ∗∗∗∗*p* < 0.0001 (unpaired two-sided *t* test; *n* > 3). Data are the mean ± SEM. *C*, immunoblot analysis of the levels of NLRP3, ASC, CASP1, phospho (P)-STAT1, STAT1, P-eIF2α, NP, NS1, and GAPDH proteins in BMDMs infected with IAV and IAV–ΔNS1. Representative blots (*n* = 2). *D*, confocal microscopy imaging of BMDMs infected with IAV or IAV–ΔNS1 (MOI 5) stained for G3BP1 to visualize stress granules and DAPI to visualize nuclei. The scale bars represent 10 μm (whole image) and 5 μm (magnified image). Representative images (*n* = 3). *E*, immunoblot analysis of CASP1 cleavage in WT and *Ifnar1*^−/−^ BMDMs infected with IAV or IAV with IFN-β supplementation (MOI 5). IFN-β was added 3 h after infection. Representative blots (*n* = 2). *F*, immunoblot analysis of CASP1 cleavage in BMDMs infected with IAV–PR8 (MOI 20) followed by arsenite (Ars), IFN-β, or Ars + IFN-β treatment. Representative blots (*n* = 3). ΔNS1, NS1 deletion mutant; IFN, interferon; IL, interleukin; PR8, Puerto Rico/8/34.

The NLRP3 inflammasome plays a critical role in limiting IAV infection-induced lung pathology in mice (14, 15). Host recognition of the RNA genome of IAV is important for mounting activation of both the type I IFN response and the NLRP3 inflammasome (5, 11, 13, 14). The IAV NS1 protein inhibits the type I IFN response to evade the host innate immune response and enable IAV replication and spread (22, 25). In the absence of NS1, IAV infection leads to a stronger type I IFN response, but the NLRP3 inflammasome is not activated. The molecular mechanism underlying the interplay between these two host antiviral responses to counteract immune evasion by IAV remains poorly understood. To address this knowledge gap, we examined how IAV infection modulated the host innate immune response in the presence and absence of NS1. First, we infected primary bone marrow–derived macrophages (BMDMs) from WT and *Nlrp3*^−/−^ mice with IAV/A/WSN/33/H1N1 (called IAV henceforth) and its NS1 deletion mutant (called IAV–ΔNS1 henceforth) (26). Immunoblotting for NS1 protein confirmed the lack of NS1 in IAV–ΔNS1–infected BMDMs, and levels of viral nucleoprotein (NP) indicated a reduced replication or infection rate after IAV–ΔNS1 infection compared with WT IAV infection (Fig. 1*A*). The reduced replication rate of IAV–ΔNS1 is in line with observations from the study describing the generation of the IAV–ΔNS1 strain, which also reported a reduced replication rate (26).

We then assessed the effect of the ΔNS1 mutation on immune activation. IAV infection in BMDMs induced cleavage of caspase-1 (CASP1), which is a measure of IAV-induced NLRP3 inflammasome activation. This cleavage was observed in WT BMDMs but not in *Nlrp3*^−/−^ BMDMs, suggesting activation of the NLRP3 inflammasome specifically (Fig. 1*A*). IAV–ΔNS1 infection, however, did not lead to CASP1 activation in WT BMDMs, suggesting that the lack of NS1 protein in IAV abolished activation of the NLRP3 inflammasome (Fig. 1*A*). Release of NLRP3 inflammasome-dependent proinflammatory cytokines, interleukin (IL)-1β and IL-18, was also significantly reduced in IAV–ΔNS1–infected WT BMDMs compared with IAV-infected cells (Fig. 1*B*). This further indicates that the deletion of NS1 results in a loss of NLRP3 inflammasome activation in BMDMs.

The protein expression levels of inflammasome components (NLRP3 and apoptosis-associated speck-like protein containing a caspase recruitment domain [ASC]) were comparable in BMDMs infected with IAV and IAV–ΔNS1, suggesting that reduced NLRP3 activation in IAV–ΔNS1–infected BMDMs was not due to defects in priming of the inflammasome (Fig. 1*C*). To further investigate the role of priming in the reduced NLRP3 activation in response to IAV–ΔNS1, we infected BMDMs with IAV–ΔNS1 and then stimulated the BMDMs with TLR ligands (Pam3Csk4 or poly(I:C)) or IFN-β. None of these stimulations rescued NLRP3 inflammasome activation in IAV–ΔNS1–infected cells (Fig. S1*A*), which further suggests that inhibition of NLRP3 activation by IAV–ΔNS1 was not due to defects in the priming of inflammasome activation. Finally, we used IAV infection as the priming signal followed by ATP or nigericin treatment for NLRP3 inflammasome activation to test the effect of NS1 loss (Fig. S1, *B* and *C*). We observed decreased CASP1 cleavage in IAV–ΔNS1–infected BMDMs, suggesting that loss of NS1 was inhibiting the NLRP3 inflammasome even with canonical triggers. In addition, the IAV NS1 protein is a robust type I IFN antagonist (22). IAV–ΔNS1 infection in BMDMs induced elevated IFN-β secretion compared with that induced by IAV, further confirming the role of NS1 in restricting type I IFN responses (Fig. 1*B*). These results indicate that the expression of NS1 promotes both IAV replication and NLRP3 inflammasome activation and inhibits the type I IFN response.

### Type I IFNs and SGs show independent regulation of IAV-induced NLRP3 inflammasome activation

SGs are membraneless cytoplasmic aggregates of RNA–protein complexes which arrest translation and also carry out other cellular functions during stress conditions (20, 27). SGs play an important role in virus–host interactions by driving antiviral responses or promoting viral replication in some cases (20, 28). IAV infection efficiently inhibits formation of SGs *via* its NS1 protein to promote translation of viral mRNAs (19, 20, 21). Specifically, the IAV NS1 protein inhibits dsRNA binding by protein kinase R and further phosphorylation of eIF2α (P-eIF2α), which are essential for SG assembly (20). We observed increased amounts of P-eIF2α and robust induction of SGs in BMDMs infected with IAV–ΔNS1 compared with WT IAV infection (Fig. 1, *C* and *D*). In addition, the dramatic increase in NP expression observed with WT IAV from 6 to 9 h after infection was much less notable with IAV–ΔNS1, suggesting that in addition to the replication defect of this virus, SGs were also exerting antiviral effects (Fig. 1*C*). Phosphorylation of signal transducer and activator of transcription 1 (STAT1), which is a measure of its activation by type I IFN signaling, was also increased in BMDMs infected with IAV–ΔNS1 compared with WT IAV, suggesting NS1 plays a role in restricting both SGs and type I IFN responses (Fig. 1*C*). However, in spite of high IFN-β levels, there was reduced NLRP3 inflammasome activation in cells infected with IAV–ΔNS1 (Fig. 1, *A* and *B*).

Previous studies have reported both inhibition and promotion of NLRP3 inflammasome activation mediated by type I IFN signaling (5, 29, 30, 31, 32). To test whether the reduced NLRP3 inflammasome activation was due to the increased production of IFN-β that occurs after IAV–ΔNS1 infection, we analyzed the effect of IFN-β supplementation on NLRP3 inflammasome activation in response to IAV infection. Exogenous IFN-β supplementation enhanced NLRP3 inflammasome activation in IAV-infected WT BMDMs (Fig. 1*E*). In addition, *Ifnar1*^−/−^ BMDMs showed reduced NLRP3 inflammasome activation both in the presence and absence of exogenous IFN-β supplementation (Fig. 1*E*). Collectively, these results suggest that the decrease in IAV-induced NLRP3 inflammasome activation in the absence of NS1 is independent of type I IFN signaling.

It is possible that IAV–ΔNS1–induced formation of intracellular SGs may play a role in NLRP3 inflammasome inhibition independent of type I IFN signaling. To investigate this possibility, we used sodium arsenite (Ars), a robust inducer of SGs that has been shown to induce SGs when added externally after IAV infection (21, 33). Ars treatment induces SGs at early and late stages of IAV infection, and IAV is less sensitive to translational arrest induced by SGs at later stages of infection (21). Thus, to avoid translational defects induced by the formation of SGs and to examine the role of SGs in NLRP3 activation during IAV infection, we infected BMDMs with IAV/Puerto Rico/8/34 (PR8; H1N1) virus (called IAV–PR8 henceforth) and treated with Ars at later stages of infection (7 h after infection). We observed that Ars treatment inhibited IAV–PR8–induced NLRP3 inflammasome activation (Fig. 1*F*) and CASP1-dependent gasdermin D cleavage (Fig. S1*D*). As reported previously (34), Ars treatment also inhibited the NLRP3 inflammasome in response to LPS + ATP treatment (Fig. S1*E*). In addition, supplementation with IFN-β did not rescue IAV–PR8–induced NLRP3 inflammasome activation and the release of IL-18, suggesting SG-mediated inhibition of the NLRP3 inflammasome was independent of IFN-β (Fig. 1*F*, Fig. S2*A*). Confocal microscopy imaging confirmed that Ars treatment, but not IFN-β supplementation, induced SGs after IAV–PR8 infection, similar to the SGs we observed during IAV–ΔNS1 infection (Fig. S2*B*). These observations establish that the cytosolic assembly of SGs, independent of type I IFN signaling, inhibits the activation of the NLRP3 inflammasome in response to IAV infection.

### SG formation specifically inhibits virus-induced NLRP3 inflammasome activation

**Figure 2:**
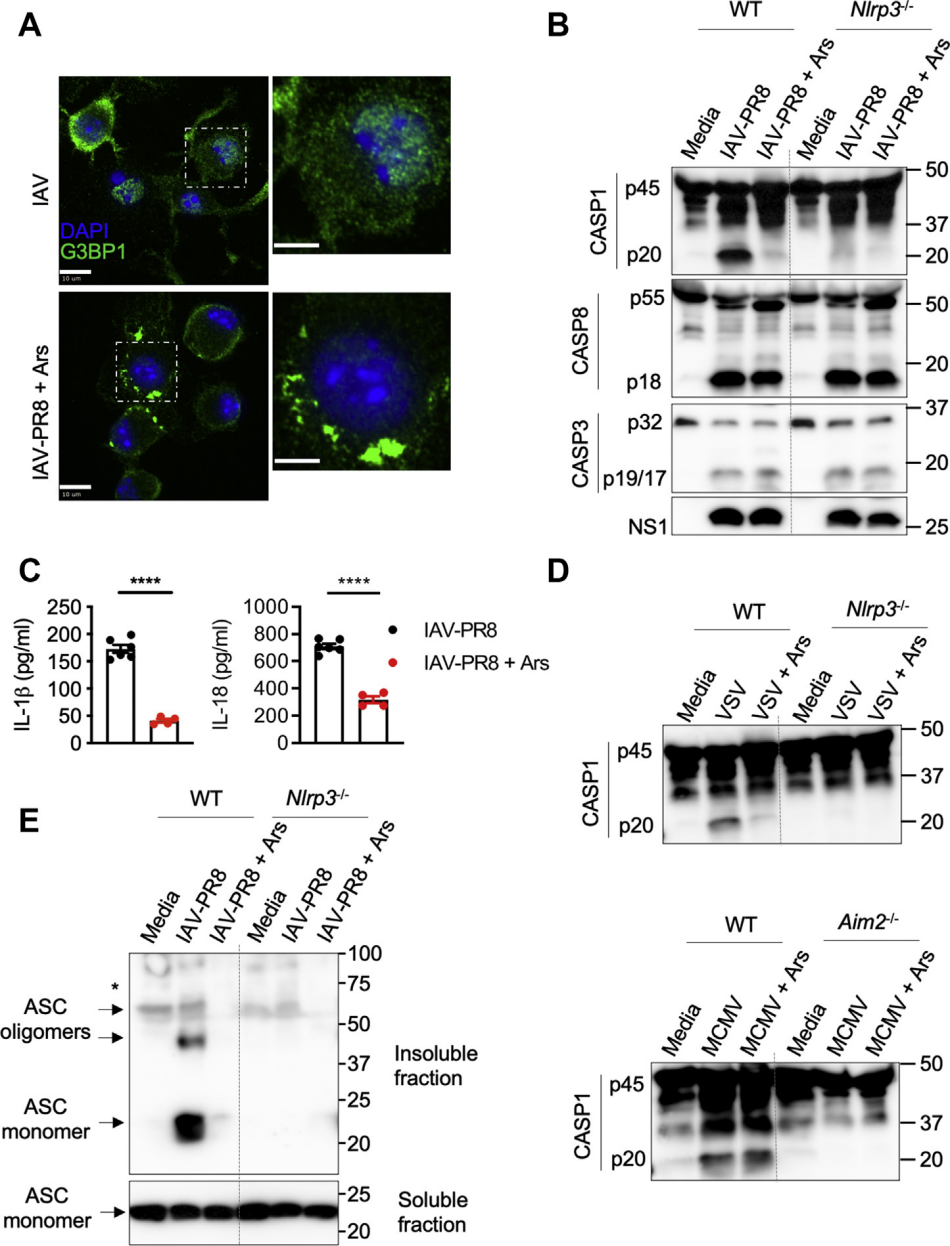
**Stress granule formation inhibits influenza-induced NLRP3 inflammasome activation by restricting its assembly.***A*, confocal microscopy imaging of bone marrow–derived macrophages (BMDMs) infected with influenza A virus (IAV)–PR8 and IAV–PR8 (MOI 20) followed by arsenite (Ars) treatment at 7 h of infection (IAV + Ars), stained for G3BP1 and DAPI. The scale bars represent 10 μm (whole image) and 5 μm (magnified image). Representative images (*n* = 3). *B*, immunoblot analysis of caspase-1 (CASP1) cleavage (pro-CASP1 (p45) and cleaved CASP1 (p20)), CASP8, and CASP3 in WT and *Nlrp3*^−/−^ BMDMs infected with IAV–PR8 or IAV–PR8 + Ars (MOI 20). Representative blots (*n* > 3). *C*, ELISA measurement of IL-1β and IL-18 in BMDMs infected with IAV–PR8 or IAV–PR8 + Ars. ∗∗∗∗*p* < 0.0001 (unpaired two-sided *t* test; *n* > 3). Data are the mean ± SEM. *D*, immunoblot analysis of CASP1 cleavage in WT and *Nlrp3*^−/−^ or *Aim2*^−/−^ BMDMs infected with vesicular stomatitis virus (VSV) or murine cytomegalovirus (MCMV) with or without Ars treatment at 7 h of infection. Representative blots (*n* = 2). *E*, immunoblot analysis of ASC oligomerization from soluble and insoluble fractions in WT and *Nlrp3*^−/−^ BMDMs infected with IAV–PR8 or IAV–PR8 + Ars (MOI 20). Representative blots (*n* = 3). The *asterisk* (∗) indicates nonspecific bands. NLRP3, nucleotide-binding oligomerization domain-like receptor with a pyrin domain 3; PR8, Puerto Rico/8/34.

To our knowledge, our observations show for the first time that SGs inhibit virus-induced NLRP3 inflammasome activation and release of proinflammatory cytokines in immune cells. We found that Ars treatment at later stages of IAV–PR8 infection induced SG formation in BMDMs (Fig. 2*A*). Ars treatment specifically inhibited IAV–PR8–induced CASP1 activation and the release of IL-1β and IL-18 (Fig. 2, *B* and *C*), but only marginally affected activation of apoptotic caspases, caspase-3 (CASP3) and caspase-8 (CASP8) (Fig. 2*B*). This suggests that Ars-induced SGs specifically inhibit the IAV-induced NLRP3 inflammasome and subsequent activation of CASP1 but do not affect apoptotic caspases. To further understand the specificity of SG-mediated inflammasome inhibition, we performed similar Ars treatment experiments in BMDMs during vesicular stomatitis virus (VSV) infection, which activates the NLRP3 inflammasome, and murine cytomegalovirus (MCMV) infection, which activates the absent in melanoma 2 inflammasome (Fig. 2*D*). We observed that Ars treatment inhibited the activation of CASP1 in response to VSV infection in BMDMs and it did not reduce the activation of CASP1 after MCMV infection (Fig. 2*D*). This further suggests that Ars-induced SGs specifically inhibit virus-induced NLRP3 inflammasome activation. This inhibition of NLRP3 activation by SGs during IAV–PR8 infection was not due to translational arrest after Ars treatment, as the protein levels of inflammasome and viral proteins were not affected (Fig. S2*C*). This indicates that SGs might also interfere with the assembly and activation of the NLRP3 inflammasome during virus infection.

To determine the effect of SGs on NLRP3 inflammasome assembly, we monitored ASC oligomerization, a measure of inflammasome assembly. We observed that Ars treatment inhibited ASC oligomerization in IAV–PR8–infected WT BMDMs (Fig. 2*E*). These findings further establish that the formation of SGs inhibits IAV-induced activation and oligomerization of the NLRP3 inflammasome.

### DDX3X is a critical regulator of host innate defense responses against IAV infection

**Figure 3:**
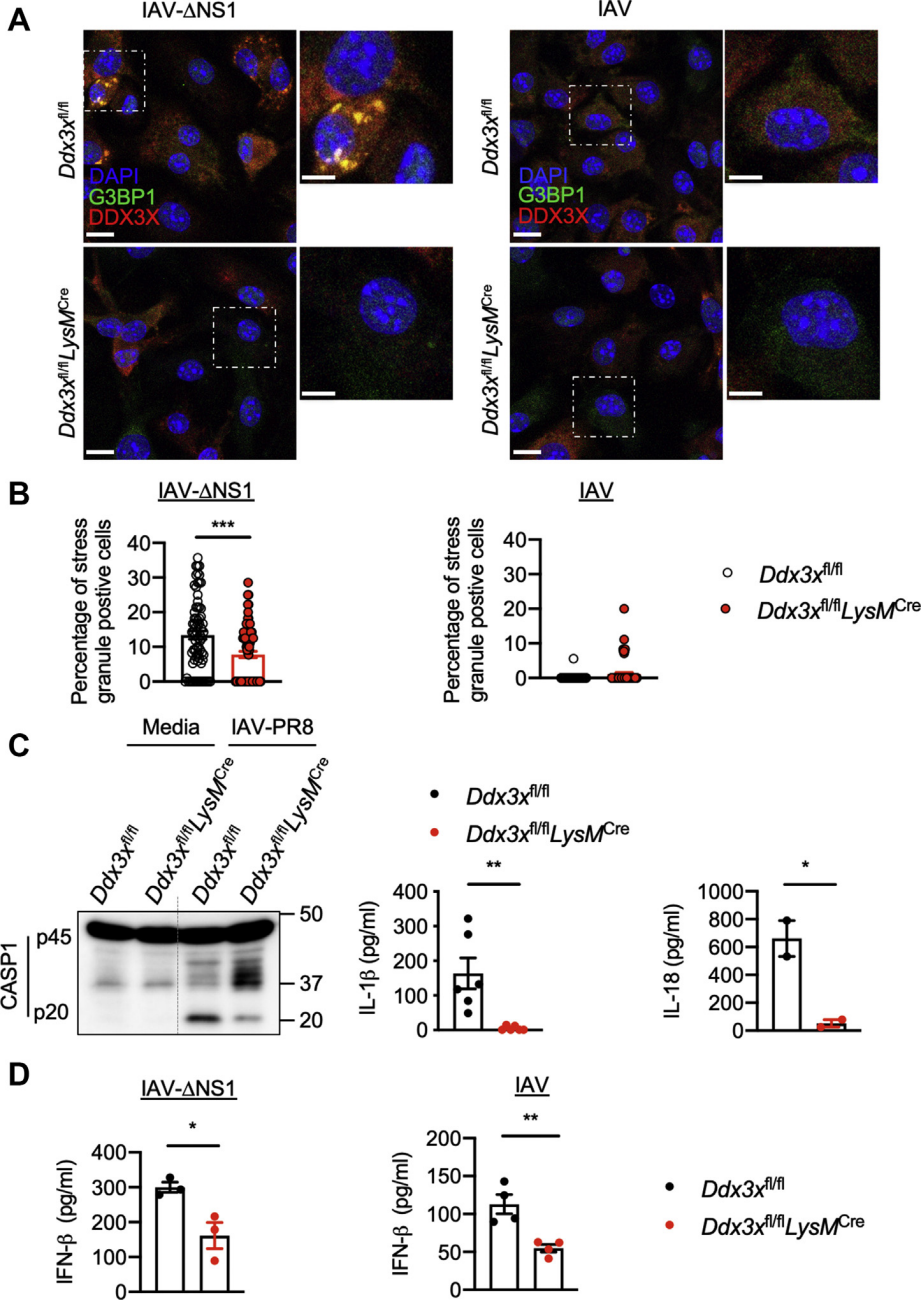
**DDX3X is a central regulator of the NLRP3 inflammasome, stress granules, and type I IFN responses during IAV infection.***A*, confocal microscopy imaging of *Ddx3x*^fl/fl^ and *Ddx3x*^fl/fl^*LysM*^Cre^ bone marrow–derived macrophages (BMDMs) infected with influenza A virus (IAV; MOI 5) or IAV–ΔNS1 (MOI 5), stained for G3BP1 and DDX3X to visualize stress granules and DAPI to visualize nuclei. The scale bars represent 10 μm (whole image) and 5 μm (magnified image). Representative images (*n* = 2). *B*, quantification of the number of cells with stress granules from confocal microscopy images. Each *circle* represents the percentage of cells in a field of view with stress granules. ∗∗∗*p* = 0.0004 (unpaired two-sided *t* test). Data are the mean ± SEM. (*n* = 2 independent experiments). *C*, immunoblot analysis of caspase-1 (CASP1) cleavage (pro-CASP1 (p45) and cleaved CASP1 (p20)) in *Ddx3x*^fl/fl^ and *Ddx3x*^fl/fl^*LysM*^Cre^ BMDMs infected with IAV–PR8 (MOI 20) and ELISA measurement of IL-1β and IL-18 in *Ddx3x*^fl/fl^ and *Ddx3x*^fl/fl^*LysM*^Cre^ BMDMs infected with IAV–PR8 (MOI 20). ∗∗*p* = 0.0054, ∗*p* = 0.0436 (unpaired two-sided *t* test; *n* ≥ 2). Data are the mean ± SEM. *D*, ELISA measurement of IFN-β in *Ddx3x*^fl/fl^ and *Ddx3x*^fl/fl^*LysM*^Cre^ BMDMs infected with IAV and IAV–ΔNS1 (MOI 5). ∗*p* = 0.0263, ∗∗*p* = 0.0054 (unpaired two-sided *t* test; *n* > 3). Data are the mean ± SEM. ΔNS1, NS1 deletion mutant; DDX3X, DEAD-box helicase 3 X-linked; G3BP1, GTPase-activating protein-binding protein 1.

RNA-binding domain–containing proteins can promote the assembly of SGs during viral infections (20, 27). DDX3X and Ras GTPase-activating protein-binding protein 1 (G3BP1) are associated with IAV-induced SG formation (18, 20, 33, 35). We indeed observed colocalization of DDX3X and G3BP1 in SGs after IAV–ΔNS1 infection (Fig. 3*A*) and also after IAV–PR8 infection followed by Ars treatment (Fig. S3*A*). To understand how SGs promote inhibition of IAV-induced NLRP3 activation, we infected BMDMs lacking *Ddx3x* expression, which were generated from a conditional KO mouse with myeloid-specific deletion of *Ddx3x* (*Ddx3x*^fl/fl^*LysM*^Cre^) (34). Lack of *Ddx3x* expression in *Ddx3x*^fl/fl^*LysM*^Cre^ BMDMs significantly reduced IAV–ΔNS1–induced formation of SGs compared with SG formation in the control *Ddx3x*^fl/fl^ BMDMs (Fig. 3*A* and *B*). Similarly, lack of *G3bp1* expression in immortalized BMDMs (*G3bp1*^−/−^ iBMDMs) reduced the formation of SGs after IAV–ΔNS1 infection (Fig. S4). This suggests that both DDX3X and G3BP1 are required for IAV–ΔNS1–induced SGs and led us to hypothesize that disrupting expression of *Ddx3x* or *G3bp1* might restore NLRP3 activation after IAV–ΔNS1 infection. However, DDX3X deficiency did not restore NLRP3 activation after IAV–ΔNS1 infection (Fig. S3*B*), and we instead observed that CASP1 cleavage was reduced in *Ddx3x*^fl/fl^*LysM*^Cre^ BMDMs compared with *Ddx3x*^fl/fl^ BMDMs when infected with IAV (Fig. 3*C*, Fig. S3*B*). Consistently, the amounts of IL-1β and IL-18 released were also reduced in IAV-infected *Ddx3x*^fl/fl^*LysM*^Cre^ BMDMs, further establishing a positive role for DDX3X in IAV-induced NLRP3 inflammasome activation (Fig. 3*C*). We also tested whether the helicase activity of DDX3X was required for activation of the NLRP3 inflammasome in response to IAV infection using a previously described inhibitor RK-33 (36). Treatment with RK-33 did not affect CASP1 cleavage, suggesting the DDX3X helicase activity is dispensable for NLRP3 inflammasome activation (Fig. S3*C*). *G3bp1*^−/−^ iBMDMs also did not show loss of IAV-induced activation of CASP1 or release of IL-1β and IL-18 (Fig. S4*B*). Although *G3bp1*^−/−^ iBMDMs formed fewer SGs after IAV–ΔNS1 infection, we still observed SGs containing DDX3X but not G3BP1 in these cells (Fig. S4*C*). These results indicate that the formation of DDX3X-specific SGs or the spatial localization of DDX3X within SGs is required to interfere with the activation of the NLRP3 inflammasome during IAV infection. This suggests that DDX3X plays an essential role in antiviral innate responses by regulating both SG-dependent type I IFN responses and NLRP3 inflammasome activation in response to IAV infection.

### DDX3X-induced SGs facilitate activation of type I IFN signaling in response to IAV infection

DDX3X primarily engaged assembly of SGs upon IAV–ΔNS1 infection and promoted NLRP3 inflammasome activation in response to NS1-expressing IAV, suggesting a mutually exclusive behavior of these host innate responses. These mutually exclusive functions of DDX3X indicate an efficient antiviral strategy for host cells which might have emerged in response to the immune evasion strategies of IAV. Formation of SGs during viral infections is associated with triggering type I IFN responses (20, 33, 35). In addition, RIG-I, which senses IAV RNA and activates type I IFN responses, localizes to IAV–ΔNS1–induced SGs in association with viral RNAs (28, 33). RIG-I–mediated phosphorylation of IRF3 (P-IRF3) is crucial for expression of type I IFNs during IAV infection (5, 11). We hypothesized that during IAV infection, DDX3X-containing SGs might act as signaling scaffolds to facilitate RIG-I activation and type I IFN production. We observed that IAV–ΔNS1–infected *Ddx3x*^fl/fl^*LysM*^Cre^ BMDMs showed a delay and substantial reduction in P-IRF3 levels (Fig. S5, *A* and *B*), indicating that DDX3X may have a role in facilitating RIG-I–mediated type I IFN responses. *Ddx3x*^fl/fl^*LysM*^Cre^ BMDMs showed a significant reduction in secretion of IFN-β and delayed activation of STAT1 (P-STAT1 levels) compared with *Ddx3x*^fl/fl^ BMDMs upon IAV and IAV–ΔNS1 infection, further establishing a role for DDX3X in IAV-induced type I IFN responses (Fig. 3*D*, Fig. S5, *A* and *B*). In addition, type I IFN signaling was abrogated in *Rigi*^−/−^ BMDMs, suggesting that DDX3X was promoting a RIG-I–mediated host response (Fig. S6, *A* and *B*). However, infection of *G3bp1*^−/−^ iBMDMs with IAV and IAV–ΔNS1 did not affect secretion of IFN-β or P-IRF3 and P-STAT1 levels compared with those of WT iBMDMs (Fig. S7, *A* and *B*). These results suggest the SG-mediated type I IFN response to IAV infection specifically requires DDX3X and RIG-I.

**Figure 4:**
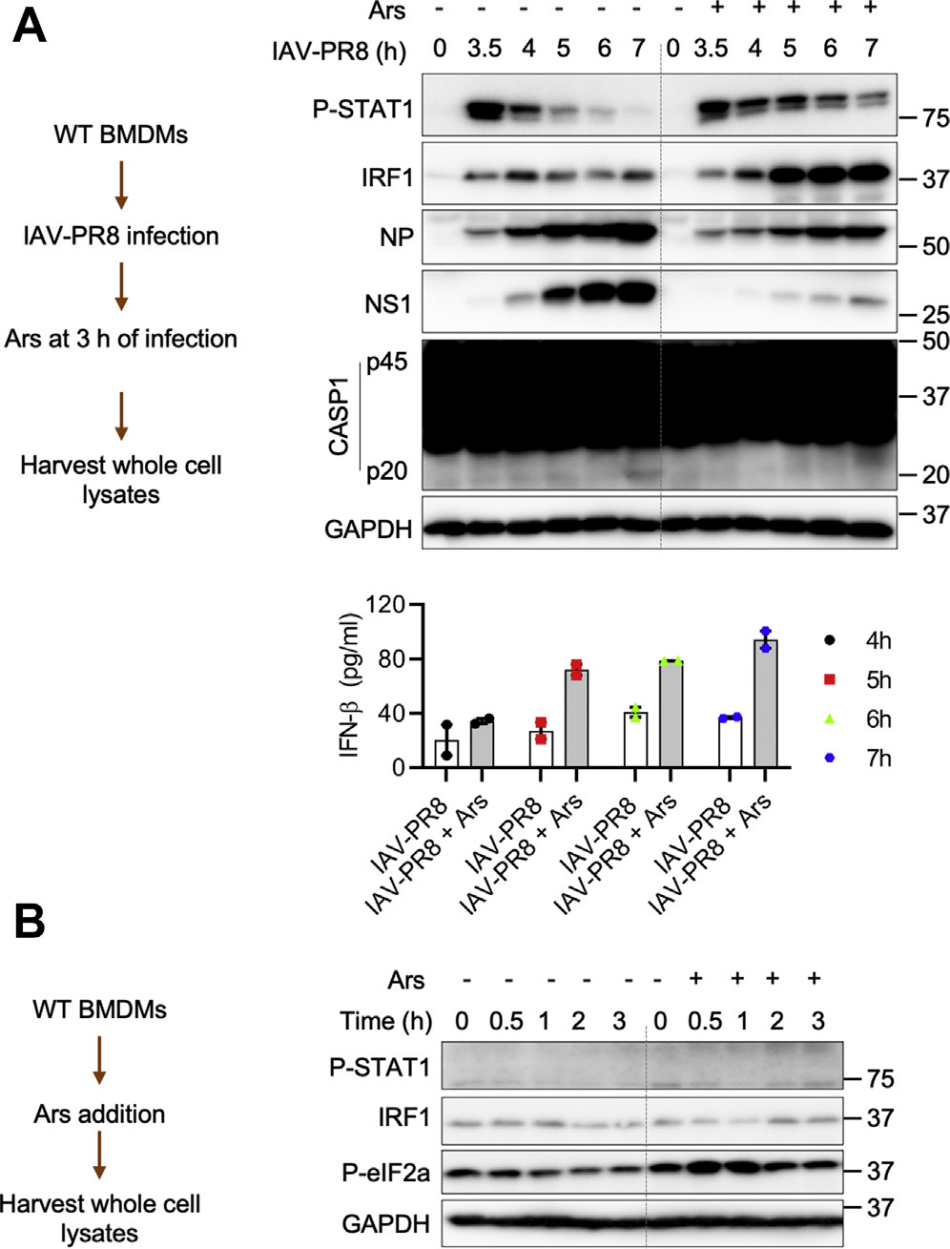
**DDX3X-induced stress granules facilitate activation of type I IFN signaling in response to IAV infection.***A*, immunoblot analysis of the levels of phospho (P)-STAT1, IFN regulatory factor 1 (IRF1), NP, NS1, caspase-1 (CASP1), and GAPDH and ELISA measurement of IFN-β in bone marrow–derived macrophages (BMDMs) infected with influenza A virus (IAV)–PR8 or IAV–PR8 (MOI 20) followed by arsenite (Ars) treatment at 3 h of infection (IAV–PR8 + Ars). Representative blots (*n* = 3). Data are the mean ± SEM in the graph. *B*, immunoblot analysis of P-STAT1, IRF1, P-eIF2α, and GAPDH in untreated or Ars-treated BMDMs. Representative blots (*n* = 2). DDX3X, DEAD-box helicase 3 X-linked; IFN, interferon; NP, nucleoprotein; NS1, nonstructural protein 1; P-eIF2α, phosphorylated eIF2α.

To further confirm whether formation of SGs facilitates RIG-I–mediated type I IFN responses, we infected BMDMs with IAV–PR8 followed by Ars treatment to induce SGs. Ars treatment after 3 h of IAV–PR8 infection increased activation of STAT1 and upregulated expression of interferon regulatory factor 1 (IRF1) (Fig. 4*A*). Ars treatment also increased the secretion of IFN-β during IAV–PR8 infection (Fig. 4*A*). A similar trend was observed when Ars was added after 5 h of IAV–PR8 infection (Fig. S8*A*). Expression of IAV proteins, NP and NS1, was reduced after Ars-induced formation of SGs and increased type I IFN signaling (Fig. 4*A*, Fig. S8). In addition, we observed reduced NLRP3 inflammasome activation after Ars treatment in IAV–PR8–infected BMDMs (Fig. 4*A*). Ars treatment alone in the absence of IAV infection did not activate STAT1 and did not induce upregulation of IRF1 (Fig. 4*B*). These results demonstrate that Ars-induced SGs enhanced IAV-induced type I IFN responses and inhibited NLRP3 inflammasome activation.

Together, these findings further establish that DDX3X is a critical factor for eliciting the antiviral innate immune response against IAV. The type of DDX3X-induced antiviral response depends on its dual ability to promote the formation of SGs and activate the NLRP3 inflammasome. The IAV NS1 protein inhibits DDX3X-mediated induction of SGs and the resulting amplification of the type I IFN response. Our results also indicate that NS1-mediated viral blockade of SGs promotes the DDX3X-driven NLRP3 inflammasome activation as a host strategy to counteract immune evasion of type I IFNs and SGs by IAV–NS1 and to facilitate antiviral immunity.

### Loss of DDX3X in the myeloid compartment increases pulmonary damage and mortality in IAV-infected mice

**Figure 5:**
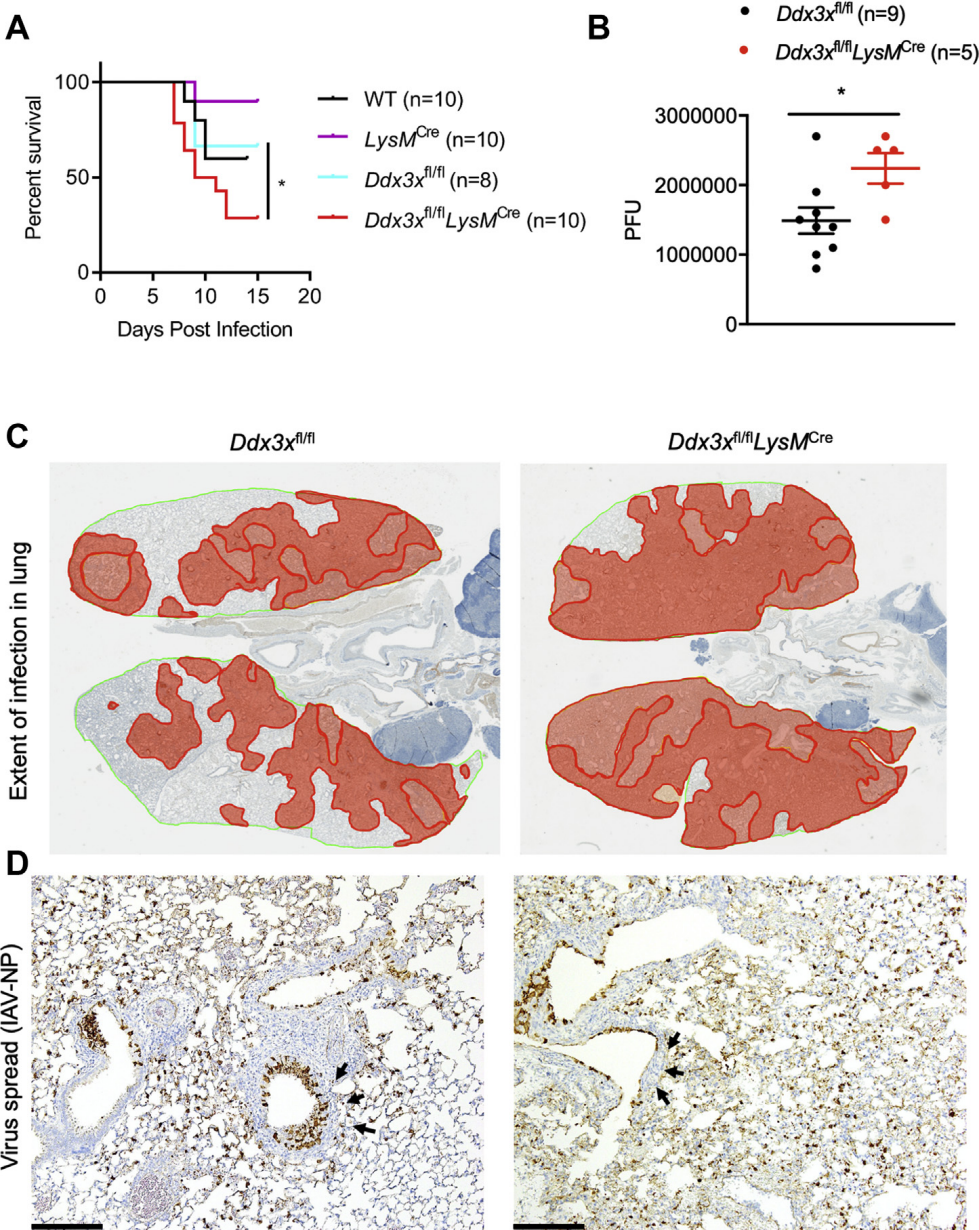
**Loss of *Ddx3x* in the myeloid compartment leads to increased susceptibility to IAV infection and tissue damage.***A*, survival analysis of WT (n = 10), *LysM*^Cre^ (*n* = 10), *Ddx3x*^fl/fl^ (*n* = 8), or *Ddx3x*^fl/fl^*LysM*^Cre^ (*n* = 10) mice to influenza A virus PR8 (IAV–PR8) infection (50 PFU). ∗*p* = 0.0137 (Mantel–Cox test). *B*, quantification of viral load in the infected lungs of *Ddx3x*^fl/fl^ (*n* = 9) and *Ddx3x*^fl/fl^*LysM*^Cre^ (*n* = 5) mice on day 5 after infection by determining the number of plaque-forming units (PFU). ∗*p* = 0.0279 (unpaired two-sided *t* test). *C*, immunohistochemistry (IHC) analysis of the infected lungs with the IAV NP antibody showing viral spread in the lungs on day 5 after infection. *D*, high-magnification images of the lungs showing increased viral load and spread and tissue damage (*black arrows*). DDX3X, DEAD-box helicase 3 X-linked; IAV, influenza A virus; NP, nucleoprotein; PR8, Puerto Rico/8/34.

Although the type I IFN response plays a critical role in resolving infection, it can also promote tissue damage by inducing a hyperactive proinflammatory cytokine response (37). Similarly, the NLRP3 inflammasome plays a protective role in the early phases of the infection and has pathogenic effects in the late stages (14, 15, 38). We found that DDX3X was critical for inducing antiviral responses, but it has also been shown to be required for viral replication, including in the context of IAV (18). Because DDX3X is important for both the host response and viral replication, we tested the effect of its loss on host survival. Complete loss of *Ddx3x* expression is embryonically lethal in mice (39, 40). Myeloid cells have previously been reported to be critical for the host response to IAV infection (41); therefore, we evaluated the effect of loss of *Ddx3x* in myeloid cells. To test the role of DDX3X in coordinating the host response to IAV infection, we infected *Ddx3x*^fl/fl^ and *Ddx3x*^fl/fl^*LysM*^Cre^ mice with 0.5 LD_50_ IAV–PR8 (∼50 PFU). *Ddx3x*^fl/fl^*LysM*^Cre^ mice showed increased susceptibility to IAV infection compared with littermate *Ddx3x*^fl/fl^ mice as measured by percent survival and viral load in the lung after IAV–PR8 infection (Fig. 5, *A* and *B*). These observations suggest that lack of DDX3X in myeloid cells leads to IAV-induced pathogenesis and compromises host survival (Fig. 5, *A* and *B*). Histological analysis of infected lungs harvested on day 5 after infection suggested that the loss of DDX3X in the myeloid compartment led to a greater extent of virus spread in the lung (Fig. 5*C*). IAV–PR8–infected lungs from *Ddx3x*^fl/fl^*LysM*^Cre^ mice had more viral particles outside the bronchiolar epithelial cells, suggesting defective clearance and uncontrolled viral spread (Fig. 5*D*). Lungs from *Ddx3x*^fl/fl^*LysM*^Cre^ mice also had denuded bronchioles, indicating severe bronchiolar epithelial loss and necrosis after IAV–PR8 infection (Fig. 5*D* and Fig. S9, *A* and *B*). These observations suggest that loss of DDX3X expression causes severe lung pathology and decreased survival during IAV infection *in vivo*. Thus, DDX3X plays a critical role in mounting protective antiviral immune responses *in vivo*.

## Discussion

IAV infection in humans and mice activates innate immune responses within 24 h of infection (8, 42). This acute activation of innate responses is necessary to create an antiviral state in the lung to help control viral load and spread. This activation is also essential to mount adaptive immune responses, which further clear viral infection and elicit immune memory to rapidly combat subsequent IAV infections (5, 6). In response to host defense activities, many RNA viruses have acquired immune evasion mechanisms to escape innate immune system–elicited restrictions and promote viral spread (11, 22). The primary function of the NS1 protein of IAV is to execute immune evasion by targeting type I IFNs, formation of SGs, and many other intracellular activities (11, 20, 22). Previous studies showed that overexpression of the influenza NS1 protein impairs NLRP3 inflammasome activation and IL-1β release and also affects ubiquitination of ASC (43, 44, 45, 46). However, our observations here indicate that infection with IAV which lacks NS1 led to the loss of NLRP3 inflammasome activation and IL-1β release, while enhancing the assembly of SGs and induction of IFN-β.

In this study, we delineated a central role for DDX3X in coordinating antiviral innate responses against IAV infection (Fig. S10). DDX3X regulates IAV-induced formation of SGs, type I IFN responses, and activation of the NLRP3 inflammasome. The NS1 protein inhibits DDX3X-mediated activation of the type I IFN response, but it leads to activation of the NLRP3 inflammasome. Loss of NS1 leads to SG assembly and more robust RIG-I–dependent type I IFN signaling. In addition, the formation of SGs restricts IAV-induced activation of the NLRP3 inflammasome. These findings imply that DDX3X might have evolved to operate distinct antiviral immune responses to counteract the immune evasion mechanisms of IAV’s NS1 protein. We observed a protective role for DDX3X against IAV-induced lung pathology *in vivo*, further illustrating the evolution of DDX3X mechanisms to counteract IAV immune evasion strategies and ultimately benefit host survival. The conserved function of DDX3X across diverse species is to regulate translation initiation and formation of SGs (18, 47, 48, 49, 50). Thus, in response to IAV infection, the original function of DDX3X may have been to induce SGs to restrict viral protein translation and mount antiviral type I IFN responses. The NS1 protein-mediated blockade of SGs and type I IFN responses might have forced DDX3X to activate other innate antiviral responses like the NLRP3 inflammasome, as we demonstrate in this study. Here we provide the first genetic evidence for the role of DDX3X in inducing host defense responses during IAV infection *in vivo*. In support of our hypothesis and findings, a recent study identified that the 1918 pandemic influenza virus promoted proteasome-dependent degradation of DDX3X to cause high virulence (51), suggesting the evolution of highly pathogenic strains to further restrict DDX3X function. These emerging findings provide a proof of principle for the mutual evolution of antiviral immune responses and viral immune evasion strategies that determine host defense responses.

Overall, our findings described here establish a critical role for DDX3X and cells of the myeloid compartment in coordinating host innate immune response to IAV infection. Our results delineate the complex interplay between type I IFN-mediated, SG-mediated, and the NLRP3 inflammasome–mediated host responses in association with immune evasion strategies of influenza viruses.

## Experimental procedures

### Mice

*Nlrp3*^−/−^ (52), *Aim2*^−/−^ (53), *Ifnar1*^−/−^ (54), *Ddx3x*^fl/fl^, and *Ddx3x*^fl/fl^*LysM*^Cre^ (34) mice have been described previously. *Ddx3x*^fl/fl^ mice were bred with *LysM*^cre+^ (*B6.129P2-Lyz2*^tm1(cre)Ifo^/J; The Jackson Laboratory) mice to generate conditional *Ddx3x*-KO mice. *Rigi*^−/−^ mice (129SvXC57BL/6XICR) were a gift from S. Akira (Osaka University, Osaka, Japan) (55). These mice were backcrossed with BALB/c mice for 10 generations to generate BALB/c-*Rigi*^−/−^ mice (31). All mice were bred at St Jude Children’s Research Hospital, and animal studies were conducted in accordance with protocols approved by the St Jude Animal Care and Use Committee.

### Cell culture

Bone marrow from mice was harvested, and primary BMDMs were cultured for 6 days in Iscove Modified Dulbecco's Medium (Gibco) supplemented with 30% L929-conditioned media, 10% fetal bovine serum (Atlanta Biologicals), 1% penicillin and streptomycin (Gibco), and 1% nonessential amino acids (Gibco). Primary BMDMs were seeded and incubated overnight before infection or treatments. *G3bp1*^−/−^ iBMDMs were generated as described previously (34).

### IAV infection and cell stimulation

The IAV–PR8 (H1N1), A/WSN/33 (WSN; H1N1; referred to as IAV), and A/WSN/33 (WSN; H1N1) ΔNS1 (referred to as IAV–ΔNS1) strains were generated by an eight-plasmid reverse genetics system as described previously (26, 56). IAV–PR8 was propagated in the allantoic cavity of 9- to 11-day-old embryonated chicken eggs. IAV and IAV–ΔNS1 were propagated in Madin-Darby Canine Kidney cells (26). Viruses were titrated by standard plaque assays.

For NLRP3 inflammasome activation experiments, BMDMs were infected with IAV–PR8, IAV, or IAV–ΔNS1 at indicated multiplicity of infection (MOI) in high-glucose Dulbecco's modified Eagle's medium (DMEM)-lacking pyruvate. Two hours after infection, the medium was replaced with high-glucose DMEM-lacking pyruvate supplemented with 10% fetal bovine serum. After overnight incubation of 15 to 18 h, lysates were prepared in caspase lysis buffer (5% NP-50, protease inhibitor cocktail (Sigma Aldrich, S8820-20TAB), 10-mM 1,4-dithiothreitol (Sigma Aldrich, 11583786001)) as described previously (34).

For signaling experiments, BMDMs were infected as described above and lysates harvested at indicated time points in RIPA buffer (protease inhibitor cocktail (Sigma Aldrich, S8820-20TAB), 150-mM sodium chloride, 1.0% NP-40, 0.5% sodium deoxycholate, 0.1% SDS, 50-mM Tris, pH 8.8).

For Ars-induced SG experiments, BMDMs were infected with IAV–PR8 (MOI 20) as described above, and 50-μM sodium (meta) Ars (Sigma) was added after 7 h of infection, and lysates were harvested after 12 h.

For RK-33 treatment, BMDMs were infected with IAV–PR8 (20 MOI) and 5-μM RK-33 (Selleckchem, S8246) was added 3.5 h after infection. Lysates were harvested 12 h after infection.

BMDMs were infected with IAV and IAV–ΔNS1 (MOI 5) for virus-induced SG experiments as described above. Cells were lysed and subjected to immunoblotting analysis.

For TLR or IFN stimulation experiments, 100 U/ml of IFN-β, 1 μg/ml of Pam3Csk4, or 10 μg/ml of poly(I:C) was added to BMDMs after 2 h of IAV infection.

For IAV + ATP and IAV + nigericin experiments, cells were infected with 5 MOI of IAV or IAV–ΔNS1 for 9 h for priming. As a positive control, BMDMs were primed with 100 ng/ml ultrapure LPS from *Salmonella minnesota* R595 (InvivoGen, tlrl-smlps) for 4 h. BMDMs were then treated with 5-mM ATP (Roche, 10127531001) or 20-μM nigericin (Cayman Chemical, 11437) for 1 h to activate the NLRP3 inflammasome.

For MCMV and VSV infections, the stock of WT-MCMV (a gift from Dr Edward S Mocarski, Emory University of School of Medicine) was prepared by infecting NIH3T3 cells at an MOI of 0.001, and the viral titer was measured by plaque assay in NIH3T3 cells. The stock of VSV (Indiana strain; a gift from Michael A Whitt, University of Tennessee Health Science Center) was prepared by infection of Vero cells at an MOI of 0.01. Viral titer was measured by plaque assay in Vero cells. MOI 10 for MCMV infection and MOI 1 for VSV infection was used for infection in BMDMs. For LPS + ATP experiment, BMDMs were primed with 100 ng/ml for 4 h, stimulated with 50-μM sodium Ars for 30 min, and followed by addition of 5-mM ATP. Lysates were harvested 45 min after addition of ATP.

### Immunoblotting analysis

Immunoblotting of whole-cell lysates and detection of specific proteins was performed as described previously (31). After infection or stimulations, BMDMs were washed with PBS, cells were lysed in RIPA buffer followed by boiling them after adding the sample loading buffer containing SDS and 2-mercaptoethanol. For CASP1 cleavage analysis, BMDMs were lysed by addition of caspase lysis buffer (5% NP-40, 10-mM dithiothreitol) (Sigma Aldrich, D9779-5G) directly to cells and media and processed as described above. Protein samples were separated on 8% or 12% polyacrylamide gels and then transferred onto PVDF membranes (Millipore). Membranes were blocked in 5% skimmed milk and incubated with the indicated primary antibodies overnight and subsequently with horseradish peroxidase (HRP)-conjugated secondary antibodies. Primary antibodies used in this study were anti-CASP1 (AG-20B-0042, AdipoGen), anti-NLRP3 (Adipogen, AG-20B-0014), anti-ASC (Adipogen, AG-25B-006-C100), anti-DDX3X (Bethyl Laboratories, A300-474A), anti-NS1 (Santa Cruz Biotechnology, SC-130568), anti-NP (Thermo Fisher Scientific, PA5-32242), anti-M1 (Abcam, ab20910), anti-G3BP1 (Proteintech, 27299-I-AP), anti–P-IRF3 (#37829S, CST), anti-IRF1 (#8478, CST), anti–P-STAT1 (#7649, CST), anti–T-STAT1 (#14994, CST), anti–P-eIF2α (#3398, CST), anti-CASP3 (#9662, CST), anticleaved CASP3 (#9661, CST), anti-CASP8 (AG-20T-0138-C100, AdipoGen), anticleaved CASP8 (#8592, CST), anti–RIG-I (#3743, CST), anti-gasdermin D (Abcam, ab209845), and anti-GAPDH (#5174, CST). HRP-conjugated secondary antibodies (anti-rabbit [111-035-047], anti-mouse [315-035-047], Jackson Immuno Research Laboratories) were used. The protein detection on membranes was performed by using Luminata Forte Western HRP Substrate (Millipore, WBLUF0500).

### ASC oligomerization

ASC oligomerization by cross-linking was performed as described previously (34). Briefly, mock-treated and IAV-infected BMDMs were subjected to lysis using NP-40 lysis buffer consisting of 1-mM dithiobis(succinimidyl propionate) crosslinker (CovaChem). Soluble and insoluble fractions were separated by centrifuging whole-cell lysates. The soluble fraction was collected, and the insoluble fraction in the form of the pellet was washed again with NP-40 lysis buffer and mixed with the sample buffer without β-mercaptoethanol. Both soluble and insoluble fractions were subjected to SDS-PAGE and immunoblotting analysis to resolve ASC oligomers.

### Confocal microscopy imaging

After infection or stimulations of BMDMs, cells were fixed in 4% paraformaldehyde (ChemCruz) at room temperature (RT) for 15 min and washed with PBS. Blocking was performed in 10% normal goat serum (Sigma). To stain SG, BMDMs were stained with the following antibodies at RT for 2 h or overnight at 4 °C: anti-G3BP1 (27299-I-AP, Proteintech) and anti-DDX3X (A300-474A, Bethyl Laboratories). BMDMs were incubated with the following secondary antibodies: Alexa Fluor 488-conjugated anti-mouse IgG (R37120, Life Technologies), and Alexa Fluor 568–conjugated anti-rabbit IgG (A-11011, Life Technologies). Nuclei of BMDMs were visualized by counterstaining BMDMs with DAPI (Vecta Labs). Confocal images were acquired on a Marianas (Intelligent Imaging Innovations, Inc) or Leica SP8 (Leica Microsystems) confocal microscope.

### Cytokine analysis by ELISA

ELISAs were performed to detect cytokines by using the IL-1β kit (88-7013-88, Invitrogen), IL-18 kit (BMS618-3, Invitrogen), and LEGEND MAX IFN-β kit (439408, BioLegend) according to the manufacturer-provided protocols.

### *In vivo* IAV infection

*In vivo* infection of IAV in mice was performed as described previously (26, 29). Briefly, 7- to 8-week-old mice were anesthetized with 250 mg/kg of Avertin and infected intranasally with 0.5 LD_50_ of mouse-adapted A/Puerto Rico/8/34 (PR8; H1N1) in 50 μl PBS. Mice were weighed daily and tracked for 16 days after infection. The humane endpoint of 30% body weight loss was used. Viral titers were determined as described previously (26, 29). Survival curve was generated in GraphPad Prism v8 software. The curves were compared using the Mantel–Cox test.

### Histopathological analysis

Lung immunohistopathologic and immunohistochemical evaluation was performed by a board-certified pathologist. The lungs from IAV–PR8–infected mice on day 5 after infection were inflated and fixed *via* intratracheal infusion with 10% buffered formalin solution. Tissues were paraffin-embedded, sectioned, and stained for virus using a primary goat polyclonal antibody (US Biological) against IAV, USSR (H1N1) at 1:1000 and a secondary biotinylated donkey anti-goat antibody (catalog number sc-2042; Santa Cruz Biotechnology) at 1:200 on tissue sections subjected to antigen retrieval for 30 min at 98 °C. The extent of virus spread was quantified by first capturing digital images of whole-lung sections stained for viral antigen by using an Aperio ScanScope XT Slide Scanner (Aperio Technologies) and then manually outlining fields with areas containing the viral antigen. The percentage of each lung field with infection/lesions was calculated using the Aperio ImageScope software.

### Statistical analysis

Statistical significance of the data was determined by the unpaired two-tailed *t* test or one-way and two-way ANOVA methods as indicated in the figure legends. The mean and error bars represent the SEM. GraphPad Prism v8 software was used for statistical analysis.

## Data availability

All data generated for this study are included within this article.

## Supporting information

This article contains supporting information.

## Conflict of interest

The authors declare that they have no conflicts of interest with the contents of this article.
