# Supplementary material for: DDX3X coordinates host defense against influenza virus by activating the NLRP3 inflammasome and type I interferon response
Source: J Biol Chem. 2021 Mar 23;296:100579. doi: 10.1016/j.jbc.2021.100579 (PMC8081917; doi:10.1016/j.jbc.2021.100579)
Supplement: Supplemental Figures S1–S10 [file mmc1.pdf]

Supplemental Figures

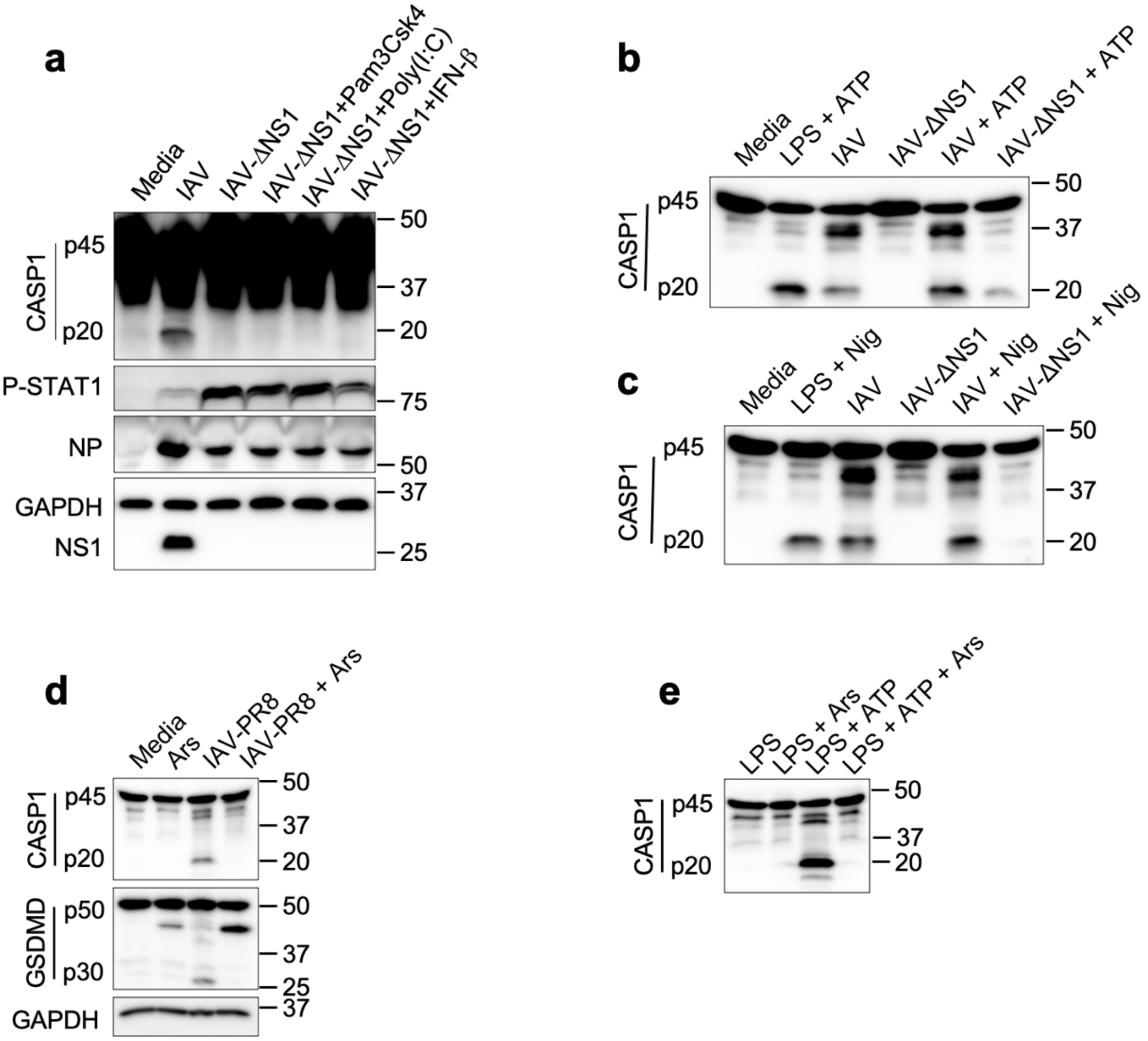

Supplementary Figure 1: Lack of NS1 inhibits IAV-induced NLRP3 inflammasome activation in a priming-independent manner

a, Immunoblot analysis of CASP1 cleavage and levels of phospho (P)-STAT1, NP, NS1, and GAPDH in bone marrow-derived macrophages (BMDMs) infected with influenza A virus (IAV), IAV-ΔNS1, or IAV-ΔNS1 followed by treatment with Pam3Csk4, poly(I:C), or IFN-β (MOI 5). Representative blots (*n* = 2). **b**, Western blot analysis of caspase-1 (CASP1) cleavage (pro-CASP1 (p45) and cleaved CASP1 (p20)) in BMDMs infected with IAV or IAV-ΔNS1 (MOI 5) for 9 hours and treated with 5 mM ATP for 1 hour to activate the NLRP3 inflammasome. Representative blot (*n* = 3). **c**, Western blot analysis of CASP1 cleavage in BMDMs infected with IAV or IAV-ΔNS1 (MOI 5) for 9 hours and treated with 20 μM nigericin (Nig) for 1 hour to activate the NLRP3 inflammasome. Representative blot (*n* = 3). **d**, Western blot analysis of CASP1 cleavage and gasdermin D (GSDMD) cleavage (pro-GSDMD (p50) and cleaved GSDMD (p30)) in response to IAV-PR8 infection (MOI 20) with or without 50 μM arsenite treatment (Ars). Ars was added 7 hours post-infection. Representative blots (*n* = 2). **e**, Western blot analysis of CASP1 cleavage in BMDMs in response to LPS + ATP treatment after stress granule induction with 50 μM Ars. Representative blot (*n* = 2).

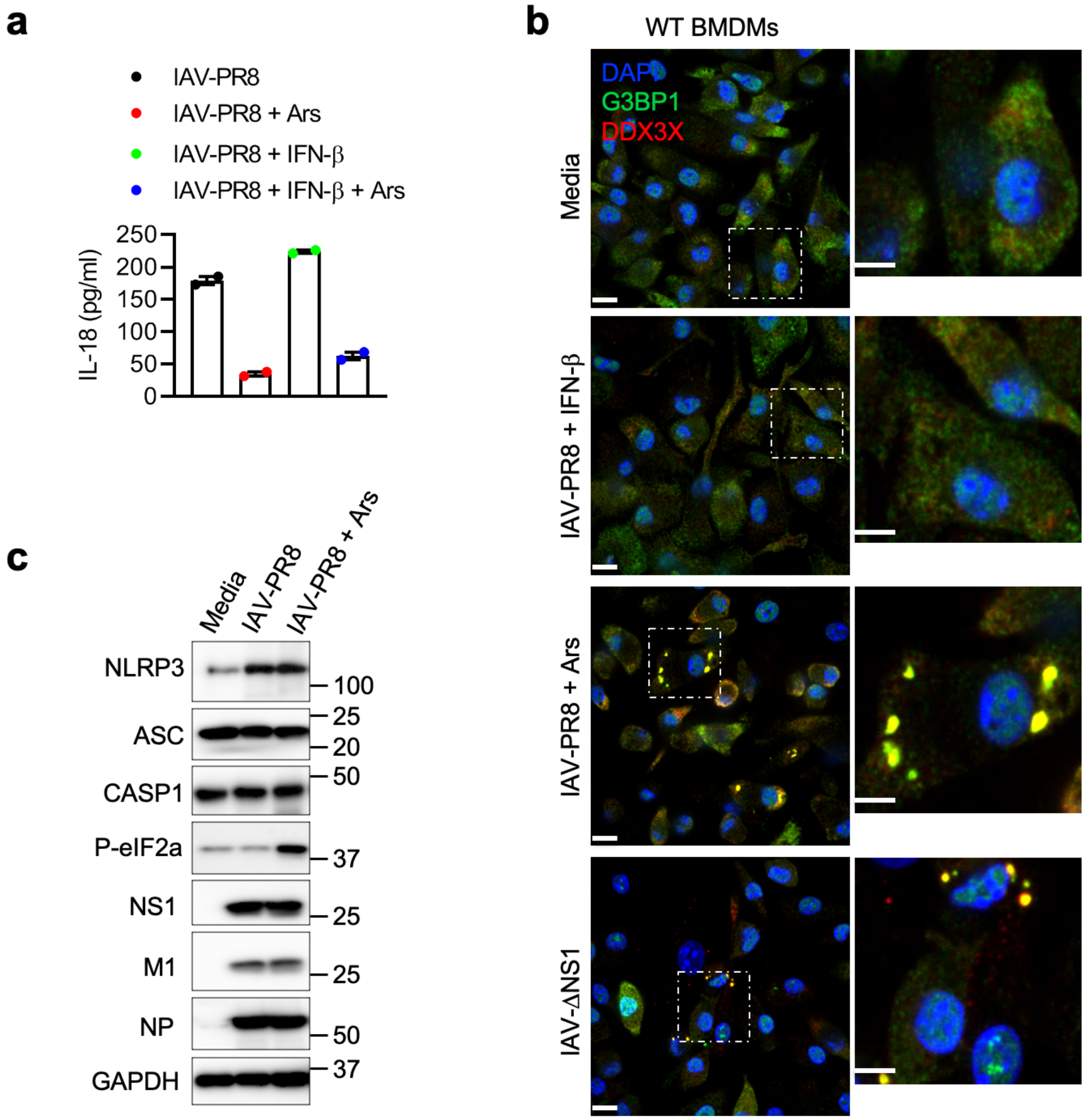

Supplementary Figure 2: Role of IFN-β and sodium arsenite (Ars) in inducing stress granules and inflammasome activation

a, ELISA measurement of IL-18 cytokine release in bone marrow-derived macrophages (BMDMs) infected with influenza A virus (IAV-PR8) or IAV-PR8 + arsenite (Ars), IFN-β, or IFN-β + Ars (MOI 20). Data are mean ± s.e.m. (*n* = 2). IFN-β was added 3 hours post-infection and Ars was added 5 hours post-infection. **b**, Confocal microscopy imaging of BMDMs infected with IAV-PR8 and treated with Ars or IFN-β stained for G3BP1 and DDX3X to visualize stress granules and DAPI to visualize nuclei (MOI 20). IFN-β was added 3 hours post-infection and Ars was added 5 hours post-infection. Infection with IAV-ΔNS1 (WSN, MOI 5) was used as a positive control. Scale bars, 10 µm (whole image); 5 µm (magnified image). Representative images (*n* = 2). **c**, Immunoblot analysis of the levels of NLRP3, ASC, caspase-1 (CASP1), phospho (P)-eIF2α, NS1, M1, NP, and GAPDH proteins in BMDMs infected with IAV-PR8 or IAV-PR8 + Ars (MOI 20). Ars was added 7 hours post-infection. Representative blots (*n* = 2).

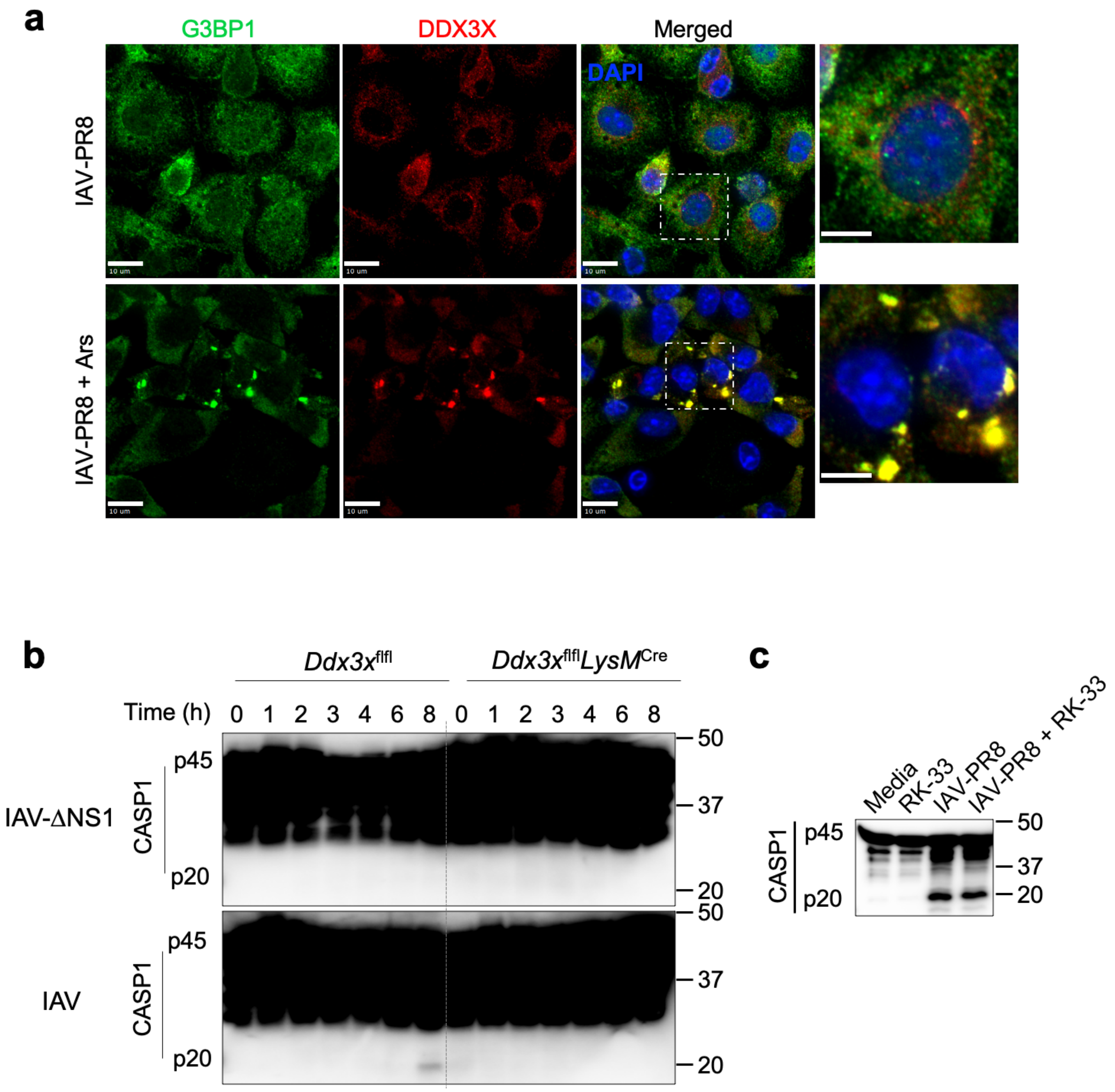

Supplementary Figure 3: IAV-induced stress granule formation and inflammasome activation require DDX3X

a, Confocal microscopy imaging of bone marrow-derived macrophages (BMDMs) infected with influenza A virus (IAV)-PR8 or IAV-PR8 + arsenite (Ars) stained for G3BP1 and DDX3X to visualize stress granules and DAPI to visualize nuclei (MOI 20). Scale bars, 10 µm (whole image); 5 µm (magnified image). Representative images (n = 2). **b,** Immunoblot analysis of caspase-1 (CASP1) cleavage (pro-CASP1 (p45) and cleaved CASP1 (p20)) in $Ddx3x^{fl/fl}$ and $Ddx3x^{fl/fl}LysM^{Cre}$ BMDMs infected with IAV or IAV-ΔNS1 (MOI 5). Representative blots ($n > 2$). **c**, Western blot analysis of CASP1 cleavage in response to IAV-PR8 infection (MOI 20) with 5 µM RK-33 treatment. RK-33 was added 3.5 hours post-infection. Representative blot (n = 2).

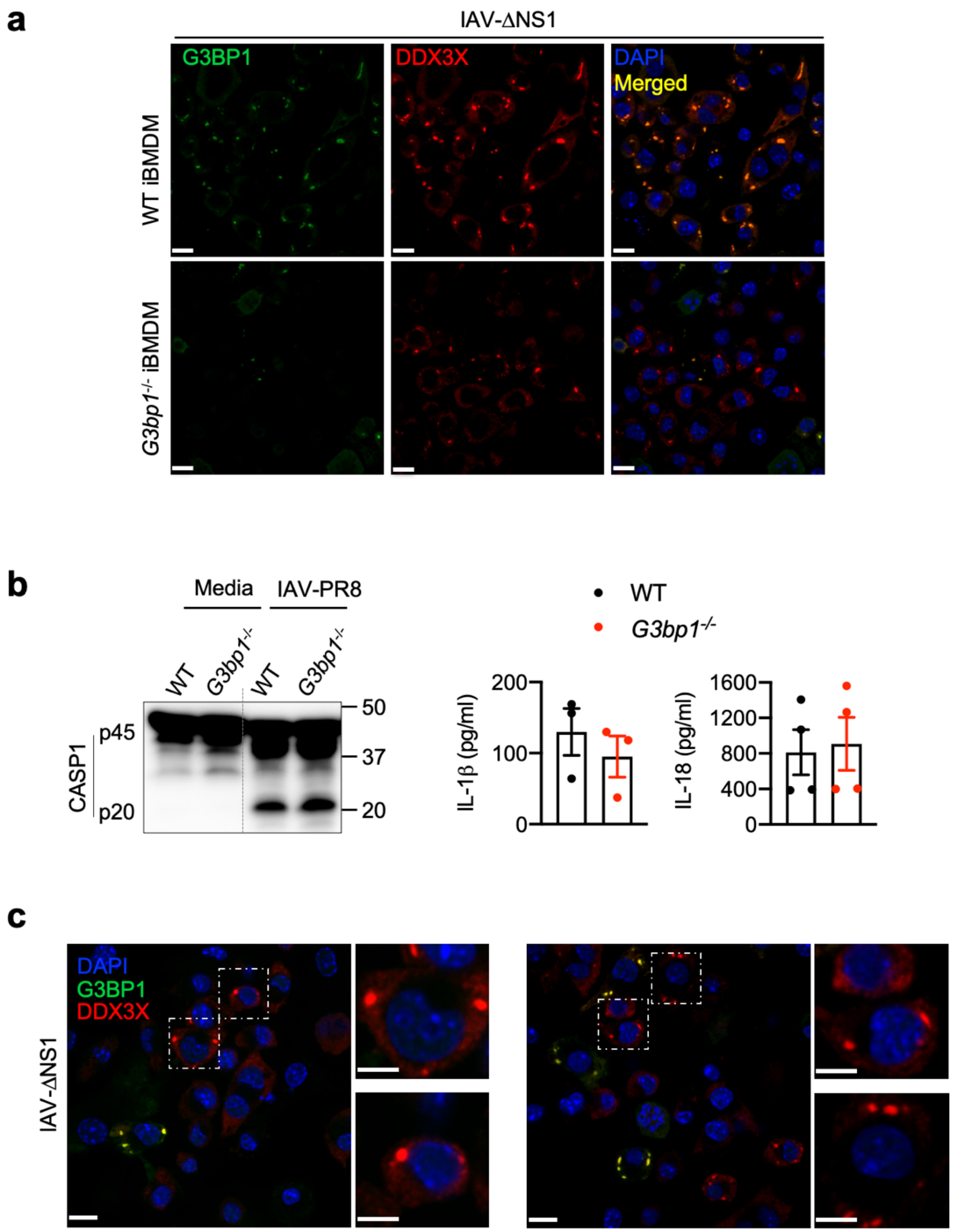

Supplementary Figure 4: Stress granule protein G3BP1 is dispensable for activation of the NLRP3 inflammasome in response to IAV infection

a, Confocal microscopy imaging of wild type (WT) and *G3bp1*$^{-/-}$ immortalized bone marrow-derived macrophages (iBMDMs) infected with influenza A virus (IAV)-ΔNS1 stained for G3BP1 and DDX3X to visualize stress granules and DAPI to visualize nuclei (MOI 20). Scale bars, 10 µm. Representative images ($n > 2$). **b**, Immunoblot analysis of caspase-1 (CASP1) cleavage in wild type (WT) and *G3bp1*$^{-/-}$ iBMDMs infected with IAV-PR8 (MOI 20) and ELISA measurement of IL-1β and IL-18. Representative blots; data are mean ± s.e.m. ($n = 2$). **c,** Confocal microscopy images of *G3bp1*$^{-/-}$ iBMDMs infected with IAV-ΔNS1 and stained for G3BP1 and DDX3X to visualize stress granules and DAPI to visualize nuclei (MOI 5). Scale bars, 10 µm (whole image); 5 µm (magnified image).

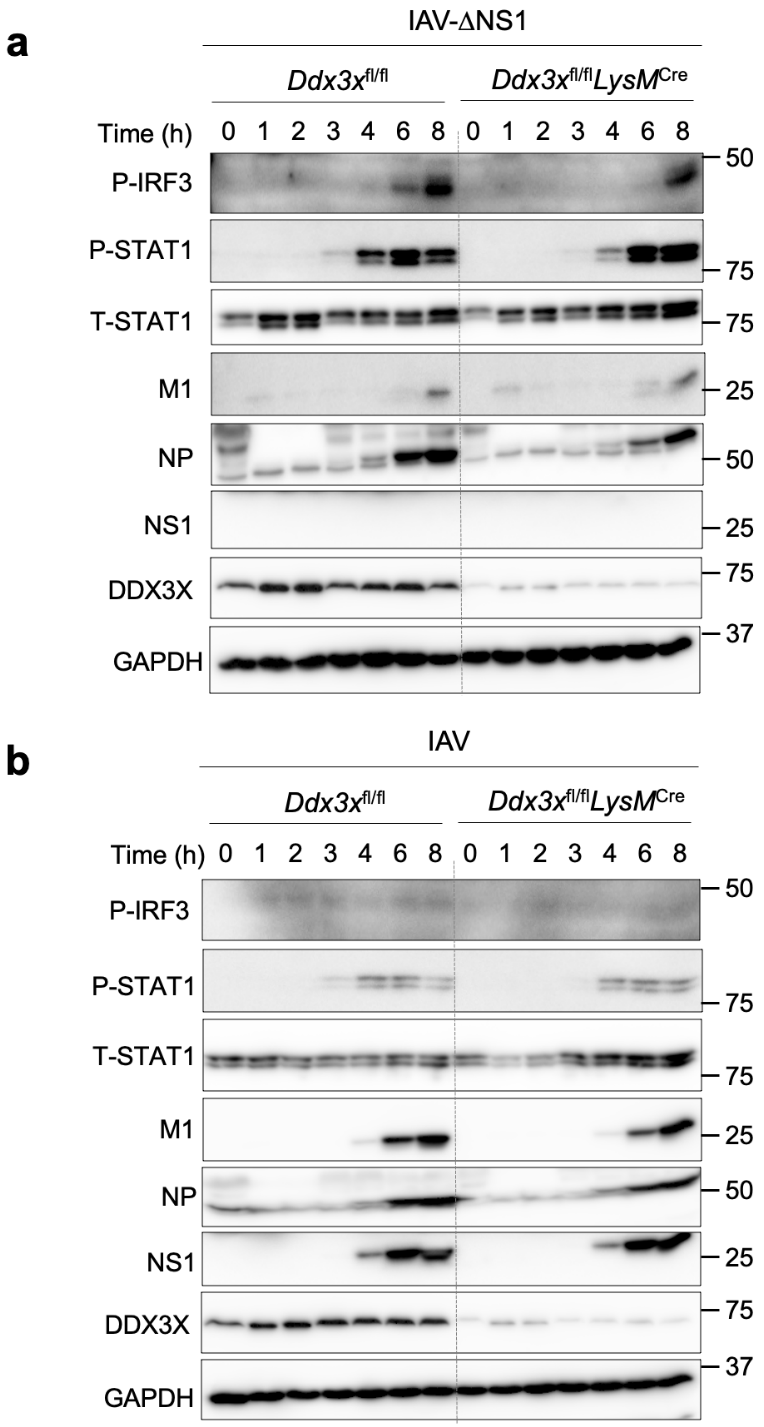

Supplementary Figure 5: Stress granule protein DDX3X is essential for IAV-induced type I IFN responses

a & b, Immunoblot analysis of the levels of phospho (P) IFN regulatory factor 3 (IRF3), P-STAT1, total STAT1, M1, NP, NS1, DDX3X, and GAPDH proteins in bone marrow-derived macrophages (BMDMs) infected with influenza A virus (IAV) or IAV-ΔNS1 (MOI 5). Representative blots ($n = 2$).

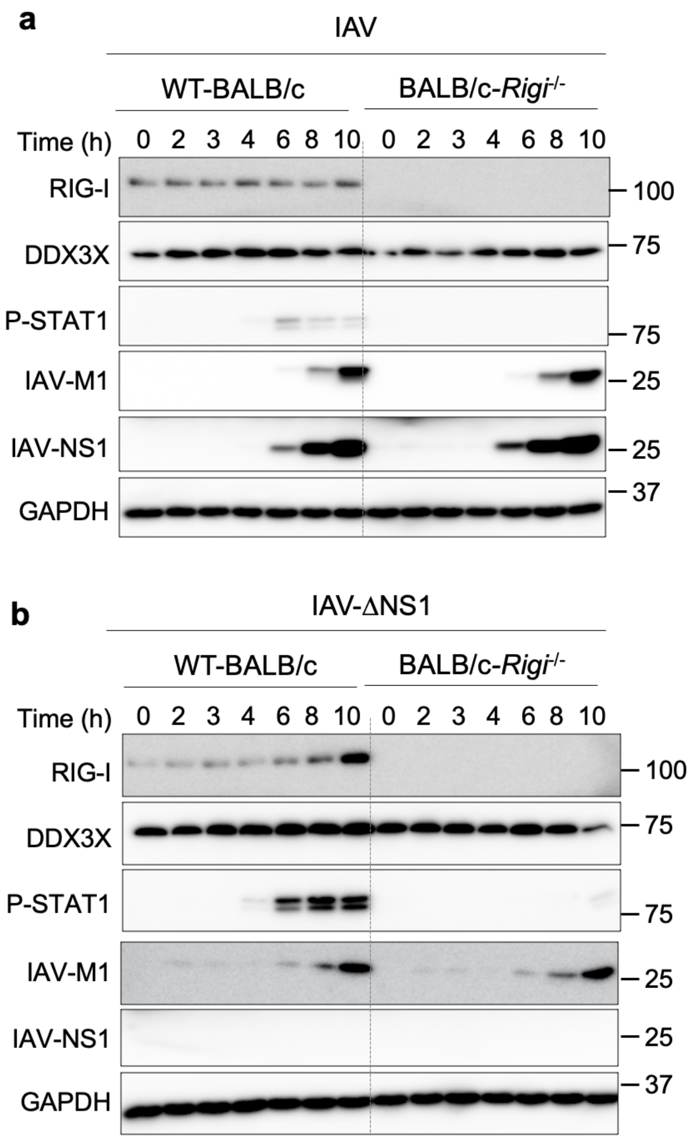

Supplementary Figure 6: Effect of *Rig-i* loss on type I interferon signaling induced by IAV and IAV-ΔNS1

a & b, Immunoblot analysis of the levels of RIG-I, DDX3X, phospho (P)-STAT1, M1, NS1, and GAPDH proteins in wild type (WT) and *Rig-i*$^{-/-}$ bone marrow-derived macrophages (BMDMs) infected with influenza A virus (IAV) or IAV-ΔNS1 (MOI 5).

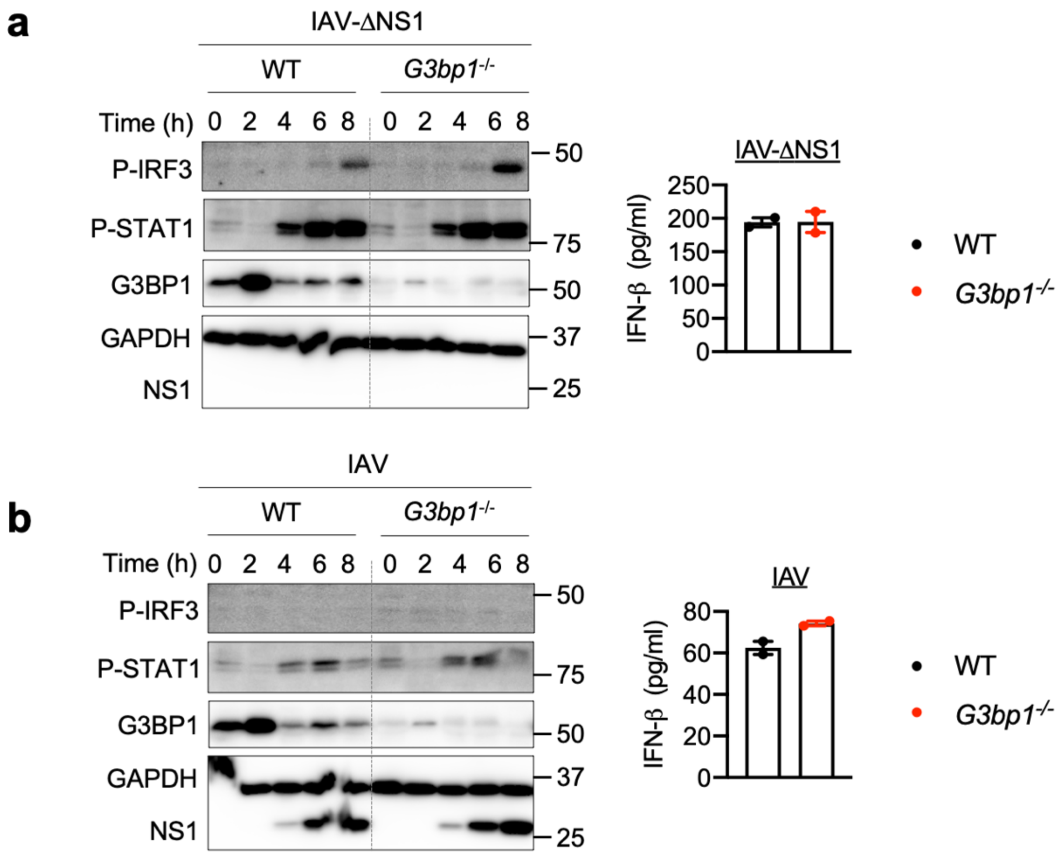

Supplementary Figure 7: Stress granule protein G3BP1 is dispensable for IAV-induced type I IFN response

a & b, Immunoblot analysis of the levels of phospho (P)-IFN regulatory factor 3 (IRF3), P-STAT1, G3BP1, NS1, and GAPDH proteins and ELISA measurement of IFN-β in wild type (WT) and *G3bp1*$^{-/-}$ immortalized bone marrow-derived macrophages (iBMDMs) infected with influenza A virus (IAV) or IAV-ΔNS1 (MOI 5). Representative blots (n = 2). Data are mean ± s.e.m. in the graphs, (n = 2).

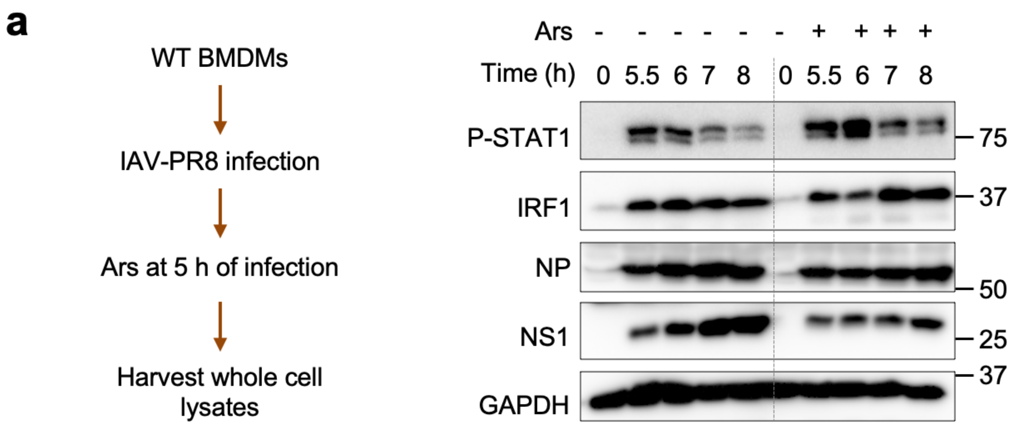

Supplementary Figure 8: Stress granule formation facilitates type I IFN signaling in response to IAV infection

a, Immunoblot analysis of the levels of phospho (P)-STAT1, IFN regulatory factor 1 (IRF1), NP, NS1, and GAPDH in bone marrow-derived macrophages (BMDMs) infected with influenza A virus (IAV)-PR8 or IAV-PR8 infection followed by arsenite (Ars) treatment at 5 h of infection (MOI 20).

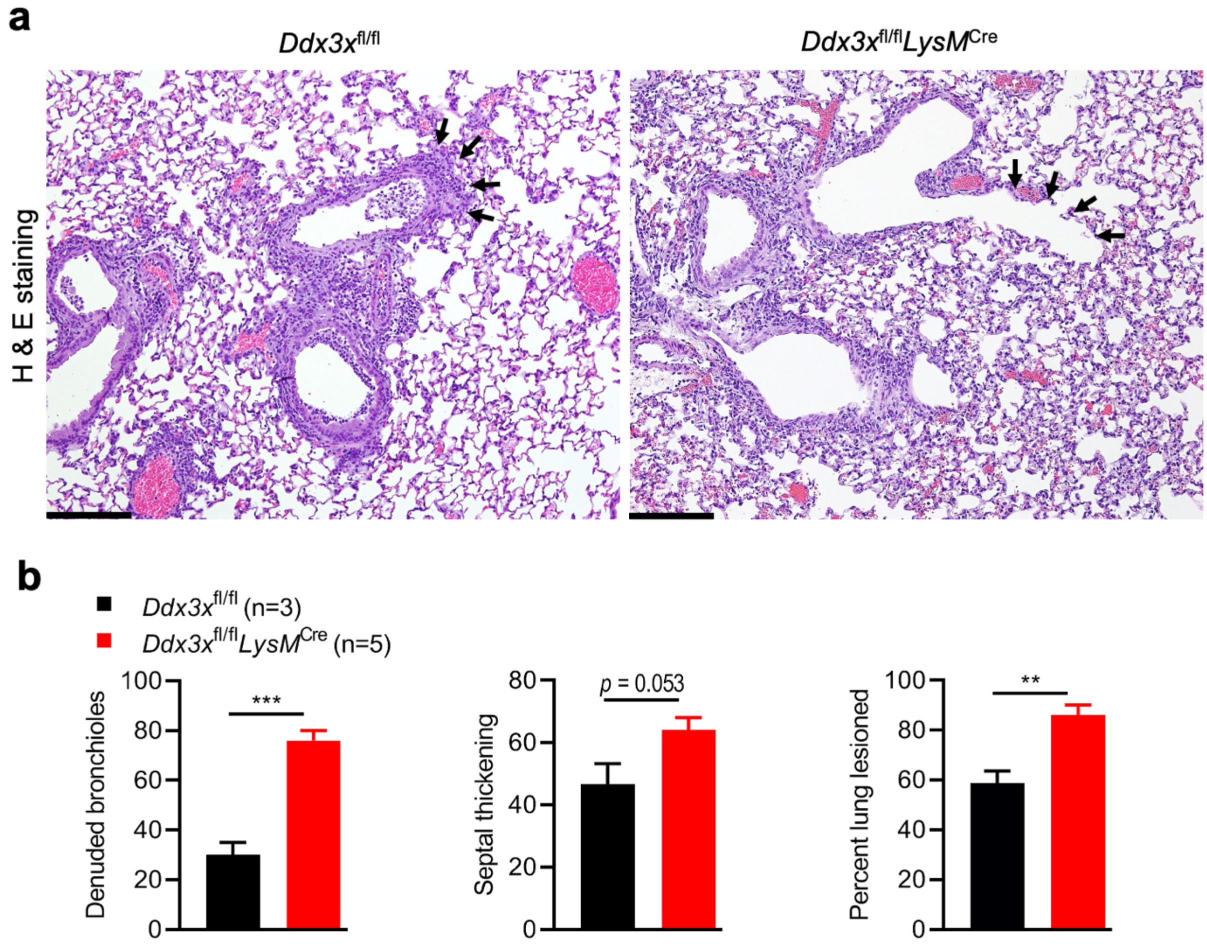

Supplementary Figure 9: Influenza infection-induced lung pathology in *Ddx3x* $^{fl/fl}$*LysM*^{Cre} mice.
a, H&E staining showing the architecture of bronchioles and inflammation. Denuded bronchioles are visible in the lungs from *Ddx3x*$^{fl/fl}$*LysM*^{Cre} mice (indicated by black arrows). **b,** Quantification of infection and tissue damage in *Ddx3x*$^{fl/fl}$ and *Ddx3x*$^{fl/fl}$*LysM*^{Cre} mice. ***P = 0.0004; **P = 0.0061 (unpaired two-sided *t*-test). Scale bars, 200 μm.

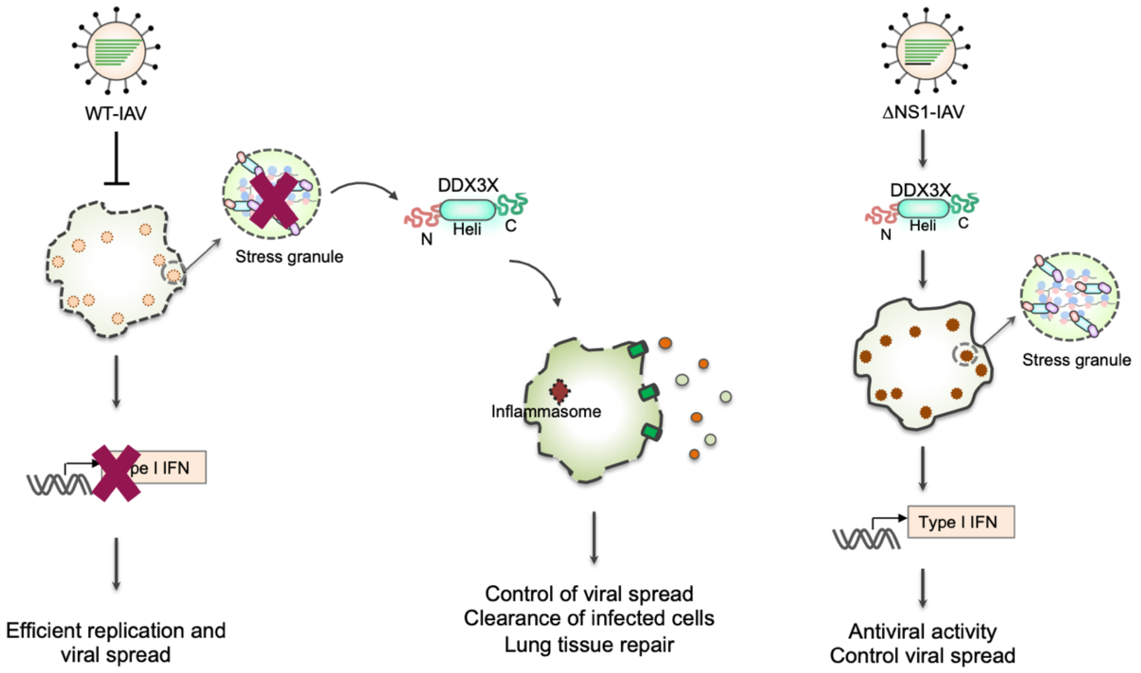

Supplementary Figure 10: Schematic of DDX3X-mediated activation of innate immune responses in response to IAV infection and its immune evasion

DDX3X promotes NLRP3 inflammasome activation in response to influenza A virus (IAV) infection where viral NS1 restricts efficient induction of type I IFN responses and formation of stress granules. Lack of NS1 in IAV promotes DDX3X-mediated stress granules and type I IFN responses.
